# The Results of Nerve-Stretching

**Published:** 1883-01

**Authors:** 


THE RESULTS OF NERVE-STRETCHING.—Dr. Ceccherelli provides a useful catalogue of 119 books and essays which treat of nerve-stretching with a summary of the history and results of the operation. The origin of the operation he traces to Billroth's treatment of a man who had bruised his right buttock severely and suffered from pain and formication in the right leg, with spasms starting from the leg, extending all over the body and endangering his life from arrest of respiration. Billroth exposed the right sciatic nerve and examined it carefully up to the sciatic notch, without finding any obvious injury, or imagining that he had done any good. The man, however, got well, and the conclusion was drawn that the manipulation of the nerve had effected his cure. Nussbaum took up the idea and made an experiment in 1872 on a man who had an abcess in the neck, which left his arm contracted and anæsthetic on its anterior surface. He laid bare the brachial plexus, and, though he found no abnormality, stretched all the component nerves strongly one by one. Some violent spasms followed, but the contracture disappeared and the sensibility returned. Since then, remarks Dr. Ceccherelli, nerve-stretching has become a part of practical surgery. Experiments show the great elasticity of nerves and their great strength. The larger trunks may be lengthened about 5 per cent., but they slowly sink again to almost exactly their original measurement. The sciatic in the dead body requires a weight of about 186 lbs. to break it [a force equal to the full strength of the grasp of a very strong hand; or, in other words, the sciatic is sufficiently strong to support a man of 13 st. weight.] Anatomically, violent stretching produces ecchymoses under the perineurium, and separation of the axis-cylinders from the neurilemma. Physiologically, it is somewhat doubtful whether the results are due to an alteration in the central or the peripheral end of the nerve, or in the substance of the nerve-trunk itself. There is no doubt that in a few instances the spinal cord is affected by traction on the nerves, as in a case of forcible reduction of the humerus after five weeks dislocation where the strain on the brachial plexus, which was matted together by inflammatory products, tore the substance of the cord and produced hæmorrhage there. The microscope throws little light on the more ordinary changes. An effect on the gray matter of the cord is sometimes argued from tropic changes which follow stretching, but these occur only when considerable violence is used. The usual result is temporary loss of sensation, without any effect on motion. Permanent loss of sensation and slight loss of motion may be produced by greater violence. Why the sensation should be the more readily affected is not plain. It is a convenient but quite unproven hypothesis that the sensory fibers are broken more easily than the motor. In surgical practice two methods are used: (1) Stretching of the nerve may be produced by very violent movements of a limb; *e.g.* Trombetta is recorded to have cured an old case of sciatica at once by flexing the hip to such an extent that the foot nearly touched the mouth: (2)

The method far more commonly in use is to expose the nerve and stretch it with the finger or some instrument. Whether it is better to draw on its central or on its peripheral end must be considered unsettled, and the force to be used to the best advantage is a matter mainly of conjecture, though most surgeons of of experience recommend more than seemed to them at first advisable.

From the large body of statistics which Dr. Ceccherelli has brought together may be constructed the following table, under hia own headings:

| | Total. | Successful. | Unsuccessful. | Deaths. | Improvement. | Doubtful. |
|---|---|---|---|---|---|---|
| Neuralgia | 99 | 74 | 6 | — | 12 | 7 |
| Contractures | 14 | 13 | — | — | 1 | — |
| Tic | 7 | 6 | 1 | — | — | — |
| Spasm from injury | 12 | 10 | 1 | — | 1 | — |
| Peripheral paralysis* | 34 | 34 | — | — | — | — |
| Diseases of nervous centers | 36 | 5 | 7 | 8 | 16 | — |
| Epilepsy | 4 | 1 | 1 | — | 2 | — |
| Tetanus | 45 | 14 | — | 29 | — | 2 |
| Stretching of optic nerve | 1 | — | — | — | 1 | — |
| Total | 252 | 157 | 16 | 37 | 33 | 9 |

The considerable excess in such dangerous cases as those of tetanus encourages Dr. Ceccherelli to recommend the treatment for a first tral. Under the diseases of nervous centers are entered the operations for relief in locomotor ataxy with the exception of sixteen recent cases of Langenbuch, in six of which he represents the pain as cured, and in the remainder the operation as too recent to justify any tabulation of the results. Benedikt throws a doubt on the ataxic nature of Langenbuch's cases, and it should be remarked that Langenbuch holds a theory of the pathology of locomotor ataxy which has few supporters (*cf.* Ricklin, *Gazette Méd. de Paris*, July 1881), viz: that it is generally started by cold, and is a peripheral lesion spreading centripetally along the nerve-trunks and by direct continuity into the posterior root-zones of the spinal cord. Bernhardt and Westphal take a decidedly

* In 30 cases a re-establishment of sensation in anæsthetic leprosy was produced by light traction on the nerves.

adverse view of the operation, saying (Feb. 1882) that they have known of no case of cure or even of decided improvement in locomotor ataxy by this means. Dr. Ceccherelli himself is inclined to favor the use of the treatment in states of such hopeless and agonizing character as occur sometimes in the course of locomotor ataxy. (*Lo Sperimentale*, Sept. 1882.)

